# Comparison of Capturing Children’s Technology Use Using Wearable Camera and Fixed Room Video: Experimental Laboratory Study

**DOI:** 10.2196/91113

**Published:** 2026-09-01

**Authors:** Amber Beynon, Charlotte Lund Rasmussen, Danica Hendry, George Thomas, Paul Davey, Juliana Zabatiero, Andrew L. Rohl, Amity Campbell, Leon Straker

**Affiliations:** 1 School of Allied Health, Curtin University, Building 401, Kent Street, Perth, Western Australia, 6102, Australia, +61 (0) 8 92661771; 2 ARC Centre of Excellence for the Digital Child, Brisbane, Queensland, Australia; 3 Health and Wellbeing Centre for Research Innovation, School of Human Movement and Nutrition Sciences, The University of Queensland, Brisbane, Queensland, Australia; 4 School of Nursing, Curtin University, Perth, Western Australia, Australia; 5 School of Electrical Engineering, Computing and Mathematical Sciences, Curtin University, Perth, Western Australia, Australia

**Keywords:** technology use, wearable camera, room video, children, screen use

## Abstract

**Background:**

Technology use by children has implications for their physical, mental, and social health and development. There are many challenges in measuring modern screen use by children. To better understand the potential impact of technology on children, it is crucial to have robust methods to measure technology use. Visual recording data may be particularly well suited to capture modern digital technology use (eg, rapid transitions, multitasking, and device switching). It would be valuable to directly compare the 2 commonly used methods (wearable camera images and room video frames) to determine what is captured best by each and how they compare when both are coded by human annotation.

**Objective:**

This study aimed to compare wearable camera images and room video frames to understand what can be captured from each source.

**Methods:**

In a laboratory study, children (aged 3‐14 years) performed various tasks while wearing a wearable camera and being video recorded by room video cameras. Comparisons between the coding of the wearable camera images and room video frames were performed on a second-by-second basis. Confusion matrices were generated to identify patterns of differences in classifications between wearable camera images and room video frame coding. Percent agreement between the wearable camera image and room video frame coding was calculated based on the confusion matrix.

**Results:**

Across 44 participants, 182,451 contemporaneous wearable camera images and room video frames were coded and merged. There were generally low levels of agreement between the codes. The highest agreement was found for “desktop” and “handheld gaming,” and the lowest agreement was found for “smartwatch.” The wearable camera and room video methods varied in sampling rate, camera movement, and field of view, leading to differences in what was captured.

**Conclusions:**

Room video might better capture technology that is within a set space and the context of the technology use, while wearable cameras could better capture active technology use, the content of technology, and different locations. Capturing technology use by children is difficult, with no gold standard and no one ideal method.

## Introduction

The impact of digital technology use by children is heavily debated, with evidence suggesting that the health effects of digital technology use may be detrimental [1,2], negligible [3], or beneficial [1,4,5]. Similarly, evidence on the educational impact of digital technology use on children is mixed [6].

One important reason for this mixed evidence is that the main measure of technology use by children is through self- or proxy-reported methods, such as questionnaires or diaries [7-9]. These subjective measures may be low cost and generally easy to administer but are often imprecise due to reporting bias and recall inaccuracy [7,8,10,11]. Another important reason is that the focus has been on just the duration of technology use (ie, screen time), despite compelling evidence that other aspects, such as device type, content, and context of use, are likely to be critical in determining the impact of technology use on child outcomes [6,12,13].

There are many challenges in measuring modern screen use by children, including children commonly using multiple devices (including those of others and sometimes simultaneously), a variety of software with differing content, for various tasks, and in a variety of contexts and locations [7]. However, to better understand the potential impact of digital technology on children, it is crucial to have robust methods to measure digital technology use. Accurately capturing important aspects of modern digital technology use other than time has been identified as a critical issue in the field [6,7].

Visual recordings have been suggested as an alternative to self- or proxy-reported methods, including the use of still photographs [14-20] or video [21] recorded on cameras worn by the child [18-20] or stationary in a room [14-16,21]. Visual recording data may be particularly well suited to capture modern digital technology use (eg, rapid transitions, multitasking, and device switching).

A camera fixed in one location can capture digital technology use within that local context, for example, within a participant’s living room. This was used in a pioneering study by Allen [14] in which time-lapsed cameras were installed in 95 families’ homes, recording images of the television 4 times per minute. This early study found differences between the recorded results and the parent-reported results in diaries. Home-based fixed room cameras have subsequently been used in several studies to capture television viewing in young children’s homes [15,16,21].

A fixed room camera may be useful if the technology use is mainly in one location (eg, a television or computer in the living room) and may create minimal burden and intrusion for participants. Still photographs (typically automatically set to capture a time-lapse sequence) or video recordings can be made from fixed-position cameras. Furthermore, depending on the camera view as well as the image clarity and resolution, it may or may not be possible to capture screen content, facial expressions, along with local context information such as coviewing [16]. However, information is limited to only activities that appear within the fixed field of view of the camera [22]. Fixed room cameras could also be intrusive to the child and family and may have third-party privacy issues [7].

Wearable cameras can be attached to participants (usually on a chest harness, headband, or a lanyard around the neck). Wearable cameras have been used to investigate different health-related behaviors in adolescents and children [18-20,23-25]. Thomas et al [19] used chest-mounted wearable cameras to capture screen use type and context in a study of 10 adolescents, and Everson et al [20] used cameras worn around the neck on a lanyard to capture screen-based activities (as well as dietary behaviors and physical activity behaviors) in a sample of 14 children.

Wearable cameras obviously move with participants; therefore, they are not restricted to one location and can thus provide location context of the digital technology use [26]. Wearing a camera may influence participant behavior, both by reminding them that their behavior is being recorded (an issue also present with fixed room recording devices) and by participant concern about damaging the device (eg, falling over and breaking the camera) or being injured by the camera (eg, if only secured on a lanyard) [20]. Many devices have limited battery life, with 1 study using wearable cameras to assess children’s dietary intake in China reporting that cameras needed to be charged twice per day [27]. Wearable cameras can be set to automatically take still images or video stream. Recordings show the field of view of the participant, which is useful. However, the view and mobility of wearable cameras have raised privacy concerns related to capturing inappropriate or unwanted images and images of third parties [28].

In addition to differences in field of view and camera movement, cameras can also vary in whether still photographs or video are recorded. Still image recording is typically done at 1 image per second or slower [17-19] sampling frequencies, whereas video recording is typically at 30 frames per second or greater [21]. Slower sampling frequencies may miss brief activities performed by children, but they also create less data to be processed. Recorded images or frames are typically coded image or frame by image or frame by humans, which is very labor intensive [7,17-19]. Slower sampling rates may also enable lower storage and battery usage. Cameras also vary in image quality, which is influenced by both resolution (pixels per image) and shutter speed. Higher pixel counts enable more detailed images, although they create larger data files. A slow shutter speed enables more light collection per image but also increases the risk of blurred images from movement of either the camera or an object in the field of view while the shutter is open.

The different aspects of visual recording options for measuring children’s use of technology appear to influence the likely information available from each method, with each method potentially being robust and valuable in its own right. Given these differences and the high processing burden in human coding of visual recordings, knowing which method would best suit a particular research project is important. It would therefore be valuable to directly compare the 2 commonly used methods (wearable camera images and room video frames) to determine what is captured best by each and how they compare when both are coded by human annotation. Furthermore, as digital technology engagement has been shown to differ between age groups [29,30] and genders [31], it would be valuable to compare whether there are any age or gender differences in what the 2 methods capture.

To our knowledge, no study has directly compared wearable camera images with room video frames within the same participants. Therefore, the aim of this study was to compare human annotation of wearable camera images and room video frames to understand what can be captured from each data source and to provide examples of issues relating to each, to help health, education, and general child development researchers decide which to use for a particular study. The secondary aim was to compare results based on children’s gender and age.

## Methods

### Study Design

An experimental laboratory study was conducted in which children performed a variety of digital technology–related and non–digital technology–related tasks while wearing a wearable camera and being video recorded by 2 room video cameras within a 10-m-diameter laboratory space.

### Participants

Typically developing children aged between 3 and 14 years were recruited among university networks and in the community via word of mouth, flyers, and social media advertisements. Recruitment was stratified based on gender and age of the participants, with the aim of recruiting approximately equal numbers of boys and girls and children in 3 age brackets according to Australian school ages (3‐5 y to represent a preschool-aged population, 6‐10 y to represent a primary school–aged population, and 11‐14 y to represent secondary school–aged and early adolescence population). Children were excluded from the study if they had any psychological or physical clinical diagnosis, as reported by their caregiver, which may have influenced their ability to understand and follow instructions or perform requested tasks.

### Ethical Considerations

Ethical approval was obtained from the Curtin University Human Ethics Committee (HRE2022-0157). Caregivers provided written informed consent, and children gave verbal or written assent, as appropriate to their age.

### Measures

#### Wearable Camera

**Figure 1.**
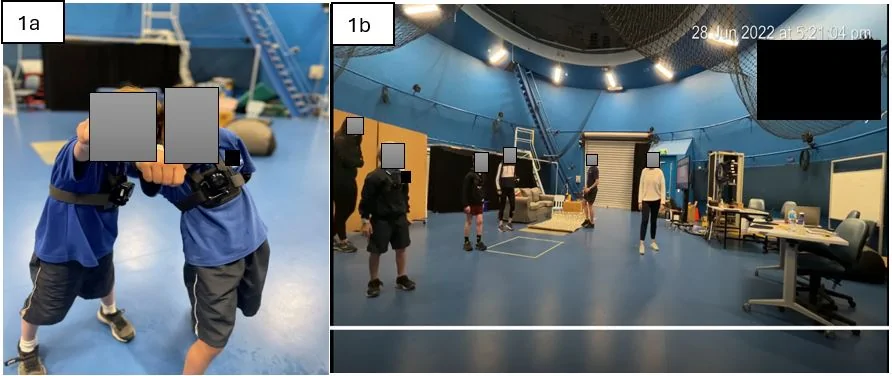
Laboratory space with cameras. (A) Children with wearable cameras. (B) Laboratory space with room video camera on tripod.

One Brinno TLC120 automatic wearable camera operating at a 1-second frequency was fitted to the child’s chest (using an adjustable chest-mounted harness to minimize the previously noted issues with lanyards; Figure 1A). The rapid transitions in digital technology use mean longer epochs risk missing brief but meaningful activities. The wearable camera weighed 101 g, was 60×60×35 mm in size, captured a 112° field of view, and did not record audio or video. Images were date- and time-stamped. The wearable camera was used to capture digital technology use during each activity directly from the field of view of the child.

#### Room Video

Room video recording was captured by 2 iPhone 12s (Apple Inc) using a super-wide lens at 30 frames per second. The iPhones were positioned on either side of the laboratory on 1-m-tall tripods approximately 4 m from the center of the room (Figure 1B). One iPhone was classified as the primary video (the most ideal location in the laboratory, providing the best view), and the second iPhone was the secondary video and was only used as a backup (eg, if the primary video battery ran out or the view was blocked by a person). The room videos were used to capture digital technology exposure during each activity by capturing the whole room.

### Study Protocol

Children attended a single approximately 1-hour data collection session at a Curtin University laboratory with their caregivers between July and October 2022. Children either participated in the data collection session alone (n=10) or with a friend or sibling (n=38), with a maximum of 2 children participating simultaneously. Two to three researchers, experienced in working with children, were present at each data collection session. After providing informed consent, caregivers completed a brief sociodemographic questionnaire. Subsequently, children were asked for their verbal or written assent, as appropriate to their age, and were then fitted with the wearable camera.

The laboratory was set up to capture different tasks, both with and without digital technology, and involving a range of postures and movements. Following laboratory and equipment familiarization, children then completed each task for at least 2 to 3 minutes, allowing for 120 to 180 images to be captured by the wearable camera (based on a 1-s epoch frequency). Tasks were performed in a standardized order where possible. However, flexibility was given in the protocol to ensure the participant’s enjoyment. Some tasks were completed with a research assistant, the child’s caregiver, or the second child in the session to replicate coviewing tasks (eg, when a child watches a television program with a caregiver or sibling) and to support children in completing the tasks. Tasks included watching programs on a television or laptop, playing on a gaming device (Nintendo Switch), reading a book, drawing on paper, playing with magnetic tiles or Jenga, using a desktop computer, drawing on a tablet, taking photographs with a smartphone, throwing and catching a ball, watching a video on a smartphone, and checking step counts via a smartwatch. Children were instructed on the position or movement to complete the task to allow the capture of images and video from a range of ecologically valid postures and movements. Table 1 provides a full list of tasks completed by the child, with devices used for each task. In total, 9 technologies were included: television, desktop, laptop, tablet, smartphone, smartwatch, handheld gaming console, combined gaming controller, and single gaming controller.

**Table 1.** Tasks completed by child.

Device	Position or movement	Details of task
Television	Sitting on bean bag	Watching a program of child’s choice on television via laptop (HDMI cable connected to television)
Television	Lying (prone)	Watching a program of child’s choice on television via laptop (HDMI cable connected to television)
Laptop	Lying (side lying)	Watching a program of child’s choice via laptop
Laptop	Sitting (chair)	Watching a program of child’s choice via laptop
Handheld gaming console	Sitting (couch)	Playing Mario Kart via Nintendo Switch using just Switch screen
Handheld gaming console	Standing	Playing Mario Kart via Nintendo Switch using just Switch screen
Book^a^	Lying (prone)	Reading a book
Stationary game (using a controller)	Sitting (couch)	Playing Mario Kart via Nintendo Switch connected to television
Magnetic tiles^a^ or Jenga	Sitting (floor)	Playing with magnetic tiles on floor
Active game	Standing while moving or jumping	Playing Just Dance or NS Sports via Nintendo Switch connected to television
Desktop	Sitting (chair)	Drawing or reading on desktop computer
Tablet	Standing	Drawing app
Pencils and crayons^a^	Standing	Drawing and coloring in with paper and colored pencils or crayons
Tablet	Standing while moving	Participating in Cosmic Kids Yoga video or similar age-appropriate movement-based YouTube video
Smartphone	Walking, jumping, running, and skipping	Taking selfies or pictures of posters while walking, jumping, running, or skipping
Ball^a^	Standing while throwing and catching	Throwing and catching a netball or basketball
Smartphone	Lying supine or crook lie (floor)	Watching “Smiling Minds” Sea Creatures video or video of choice
Smartwatch	Jumping, handstands	Jumping and handstands then checking heart rate or steps via smartwatch
Smartwatch	Running, cartwheels	Running and then checking heart rate or steps via smartwatch screen
Smartwatch	Climbing stairs	Climbing stairs and then checking heart rate or steps via smartwatch screen

a Nontechnology-based tasks.

### Analysis

#### Wearable Camera Coding

The wearable camera automatically processed images into a time-lapse video file (.avi), which was later downloaded and converted into single images (.jpg) using the open-source software FFmpeg (version 4.3 FFmpeg Team), which labeled each image with a participant code and image number. Images for each participant were viewed by a member of the research team, who recorded the corresponding technology code (Table 2) for each image recorded in a spreadsheet with image numbers and time stamps. Each image was coded in chronological order as indicated by the image number. The coder first determined if the image was codable or not. Uncodable images were images that could not be confidently annotated due to poor image quality, such as if the image was blurry, had poor lighting, or was obscured. If codable, each image was then coded to determine if any digital technology was visible. If not, then the image was coded as “no digital technology.” If a digital technology was visible and active (ie, screen turned on), then the digital technology in the image was coded first for the primary technology device (the technology related to the primary task the child was doing). If there was more than one technology device visible in the image, any other technology was also coded, allowing for up to 3 technologies to be coded for the same image.

**Table 2.** Technology code definitions.

Coding options	Definition
Uncodable: blurry	Any image or set of images where the image quality is so poor due to being blurred that the coder is unable to confidently determine what is occurring in all aspects of the image.
Uncodable: obscured images	Any image or set of images where the image quality is so poor due to lighting that the coder is unable to confidently determine what is occurring in all aspects of the image. Poor lighting can include images that are too dark or too overexposed to accurately determine anything.
Uncodable: poor lighting	Images that are completely black or fully blocked by something and cannot be coded as having an active screen-based media device according to the coding rules. Includes completely blacked images.
Unsure	Coder is uncertain what is in the image.
No digital technology	No digital technology is visible (or active for room video).
Television	A device shaped like a box or rectangle with a screen that receives electrical signals and changes them into moving images. Can stand-alone or be mounted to a wall. Can be a smart television (ie, internet connected).
Desktop computer	A computer that fits on a desk but is not easily moved from place to place. Has a monitor, keyboard, mouse, and tower.
Laptop computer	A computer that is small enough to be carried around easily and is flat when closed. Indicated by an inbuilt keyboard.
Smartphone	A handheld device that can be used as a small computer, connect to the internet, and run apps.
Tablet	A small, flat computer that is controlled by touching the screen with one’s finger or a special pen. Does not require a keyboard or mouse. Includes e-readers.
Handheld gaming console	Portable, self-contained devices that have a built-in screen, game controls, and speakers.
Combined gaming controller	When the child has the Nintendo Switch controller, which is controlled by 2 hands (ie, it has both the red and the blue parts together or the black controller in their hand).
Single gaming controller	When the child has the controller separated and/or in separate hands.
Smartwatch	A watch that has an electronic screen with features of a smartphone or a computer. Includes fitness trackers, such as a FitBit.

#### Room Video Coding

Video files were downloaded from the iPhones, with separate files for each participant. The secondary video was used for 4 participants (due to the primary video battery running out). The video frames for each participant were viewed by a member of the research team using QuickTime software (Apple Inc), with the technology code (Table 2) for each frame recorded in a spreadsheet. Video frames were coded in chronological order. To gain an understanding of the context, coders were encouraged to watch several video frames prior to coding. The coder would first determine if a frame was codable using the same codes as those used for the wearable camera images. Next, the coder would determine if any technology was visible and active. The definition of “active” varied from that used for wearable camera images, given the broader field of view of the room video, which always included screens not always in use, such as the television screen. To be an active screen, it needed to be (1) within the child’s field of view as determined by the child’s head position, (2) facing the child, and (3) turned on or the child had active engagement with the screen. For smartwatches or gaming controllers to be coded, the child had to be actively engaging with the device. As with the wearable camera images coding, the primary technology was the technology related to the primary task the child was doing, and up to 2 additional technologies could be coded if they met the abovementioned criteria and were within the video frame. A custom-made Python program was used to generate second-by-second datasets of the video code spreadsheets to enable synchronization with the wearable camera code spreadsheets using a lookup table approach, whereby each time stamp in one dataset was matched to its corresponding time stamp in the other.

#### Interrater Reliability of Wearable Camera and Fixed Room Video Coding

The wearable camera images and room video frames from 3 participants (one from each age group) were independently coded by a second researcher to determine interrater reliability by assessing percentage agreement and Cohen κ [32]. For the wearable camera images, a very good interrater reliability was observed for all age groups, with an average percentage agreement of 92.5% (range 90.3%‐94.7%) and an average κ of 0.88 (range 0.86‐0.92). For the room video frames, a good level of interrater reliability was found, with an average percentage agreement of 84.8% (range 72.7%‐98.8%) and an average κ (range 0.66‐0.98), with the lowest level of agreement found for the youngest participant.

#### Statistical Analysis

As up to 3 technologies could be coded for each wearable camera image and room video frame, a variable was created to describe all possible combinations of the 9 technologies (television, desktop, laptop, tablet, smartphone, smartwatch, handheld gaming console, combined gaming controller, and single gaming controller), resulting in a total of 50 technology combinations (eg, television and combined gaming controller being used simultaneously when playing a video game). Including “no digital technology,” “unsure,” and “uncodable” created 53 categories (Table S1 in Multimedia Appendix 1).

An additional “any technology combination” variable was created to describe when each of the technologies was coded either alone or in combination with another technology (eg, “any television” when television was coded either alone or in combination with other technologies). This resulted in a variable with a total of 9 categories: any television, any desktop, any laptop, any tablet, any smartphone, any smartwatch, any handheld gaming console, any combined gaming controller, and any single gaming controller. As the technology types could be used together simultaneously, the 9 “any technology combination” categories were not distinct categories, as each individual image or frame could be coded for a number of these categories.

Comparisons between the coding of the wearable camera images and room video frames were made on a second-by-second basis. Confusion matrices were generated for the 53 categories to identify patterns of differences in classifications between wearable camera image and room video frame coding. Percent agreement between the wearable camera image and room video frame coding was calculated based on the confusion matrix with the 53-category variable.

The 9 “any technology combination” categories were used to determine if a particular technology was coded at the same second by both the wearable camera and room video regardless of whether the technology was coded by itself or with other technologies. Percent agreement between the wearable camera image and the room video frame coding was calculated based on whether either source coded the technology. As percent agreement does not take into account chance agreement, and given the different prevalence of some technologies, prevalence-adjusted bias-adjusted κ (PABAK) was also calculated [32,33]. We interpreted PABAK values using the Landis and Koch [34] classification scale where below-chance agreement <0.00, slight agreement 0.00 to 0.20, fair agreement 0.21 to 0.40, moderate agreement 0.41 to 0.60, substantial agreement 0.61 to 0.80, and almost perfect agreement 0.81 to 1.00. Agreement between the wearable camera and room video was compared between gender and age groups using separate two-way *t* tests or Wilcoxon tests (depending on distribution of data) comparing PABAK between genders and one-way ANOVA or Kruskal-Wallis (depending on distribution of data) comparing PABAK between age groups.

## Results

### Overview

A total of 48 children completed the study; however, a technical error resulted in some missing wearable camera image time stamps. Therefore, 4 participants were excluded, leaving a sample of 44 participants (girls: n=25, 55%) with a mean age of 8.3 (SD 3.3) years (Multimedia Appendix 2). Eleven children were aged between 3 and 6 years, 22 children were aged between 7 and 10 years, and 11 children were aged between 11 and 14 years. Across the 44 participants, a total of 182,451 contemporaneous wearable camera images and room video frames were coded, which equated to 50.7 hours of observed recording. At an individual participant level, the mean recording duration was 72.5 (SD 11.2, range 39.7-103.0) minutes.

### Frequency of Technology Codes for Wearable Camera Images and Room Video Frames

Table 3 presents the frequency distribution of categories with at least 100 images or frames using the 53 categories of possible technology codes based on the wearable camera images and room video frames. Table S2 in Multimedia Appendix 1 contains the distribution of all 53 categories. “No digital technology” was coded for 46% (n=84,178) and 29% (n=52,504) of the wearable camera images and room video frames, respectively. The most frequently coded technologies based on the wearable camera images were “television” (n=21,900, 12%), “laptop” (n=11,596, 6%), and “desktop*”* (n=9426, 5%). For the room video frames, the most frequently coded technologies were “laptop” (n=19,150, 11%), “television” (n=18,266, 10%), and “television & single gaming controller” (n=16,005, 9%). Ten percent (n=17,802) of the wearable camera images were coded as “uncodable” compared to only 3 (0%) room video frames.

**Table 3.** Frequency and agreement of 53 category technology coding based on wearable camera and room video recordings.

Technology coded	Wearable camera images (N=182,451), n (%)	Room video frames (N=182,451), n (%)	Agreement, n (%)
Uncodable	17,802 (9.8)	3 (0)	0 (0)
Unsure	234 (0.1)	39 (0.02)	0 (0)
No digital technology	84,178 (46.1)	52,504 (28.8)	41,113 (48.9)
Television	21,900 (12)	18,266 (10)	3825 (17.5)
Desktop	9426 (5.2)	11,893 (6.5)	8759 (73.6)
Laptop	11,596 (6.4)	19,150 (10.5)	10,510 (54.9)
Tablet	7327 (4)	14,565 (8)	5299 (36.4)
Smartphone	4866 (2.7)	9120 (5)	3063 (33.5)
Smartwatch	182 (0.1)	1822 (1)	57 (3.1)
Handheld gaming console	6812 (3.7)	10,758 (5.9)	5988 (55.7)
Combined gaming controller	1922 (1.1)	486 (0.3)	182 (9.4)
Single gaming controller	176 (0.1)	30 (0.02)	0 (0)
Laptop and combined gaming controller	76 (0.04)	0 (0)	0 (0)
Laptop and handheld gaming console	310 (0.2)	113 (0.1)	75 (24.2)
Tablet and smartphone	6 (0)	262 (0.1)	1 (0.4)
Tablet and tablet	1111 (0.6)	0 (0)	0 (0)
Television and combined gaming controller	4142 (2.3)	9346 (5.1)	3028 (32.4)
Television and laptop	6608 (3.6)	9970 (5.5)	4068 (40.8)
Television and single gaming controller	2034 (1.1)	16,005 (8.8)	1559 (9.7)
Television and smartphone	460 (0.3)	4117 (2.3)	150 (3.6)
Television and smartwatch	64 (0.04)	432 (0.2)	8 (1.8)
Television and tablet	389 (0.2)	3147 (1.7)	155 (5)
Television and laptop and smartwatch	0 (0)	171 (0.09)	0 (0)
Television and single gaming controller and single gaming controller	286 (0.2)	0 (0)	0 (0)

Table 4 presents the frequency distribution of categories using the 9 “any technology combination” category codes for both the wearable camera images and room video frames. The most frequently coded technologies based on the wearable camera images were “any television” (36,198/182,451, 19.8%), “any laptop” (n=18,816, 10.3%), and “any desktop” (n=9479, 5.2%). For the room video frames, the most frequently coded technologies were “any television” (n=61,477, 33.7%), “any laptop” (n=29,461, 16.1%), and “any tablet” (n=18,075, 9.9%)*.*

**Table 4.** Frequency and agreement of “any technology combination” coding based on wearable camera images and room video frames.

Technology	Wearable camera images (N=182,451), n (%)	Room video frames (N=182,451), n (%)	Total (N^a^)	Agreement, n (%)	PABAK^b^
Any television	36,198 (19.8)	61,477 (33.7)	67,617	30,058 (44.5)	−0.111
Any desktop	9479 (5.2)	11,933 (6.5)	12,628	8784 (69.6)	0.391
Any laptop	18,816 (10.3)	29,461 (16.1)	33,077	15,200 (46)	−0.081
Any tablet	8904 (4.9)	18,075 (9.9)	19,150	7829 (40.9)	−0.182
Any smartphone	5480 (3)	13,564 (7.4)	14,288	4756 (33.3)	−0.334
Any smartwatch	246 (0.1)	2661 (1.5)	2821	86 (3)	−0.939
Any handheld gaming console	7154 (3.9)	10,865 (5.9)	11,705	6314 (53.9)	0.079
Any combined gaming controller	6314 (3.5)	9829 (5.4)	11,155	4988 (44.7)	−0.106
Any single gaming controller	2642 (1.4)	16,033 (8.8)	16,792	1883 (11.2)	−0.776

a Coded by either wearable camera and/or room video.

b PABAK: prevalence-adjusted bias-adjusted κ.

### Comparison of Technology Codes From Wearable Camera Images and Room Video Frames

#### Overview

Table 3 presents the agreement of the 53-category codes from the wearable camera images and room video frames. Table 4 presents the agreement of all possible classifications for each general technology type combined (ie, the 9 “any technology combination”) codes from the wearable camera images and room video frames. Regardless of whether technology was coded uniquely (53 codes) or in any combination (9 codes), there was generally below-chance agreement between the wearable camera image–based and room video frame–based codes. Specifically, for the 9 “any technology combination,” the average percent agreement was 38.6% (range 3%‐69.6%), with PABAK scores ranging from −0.939 to 0.391.

While the study did not have an independent reference standard to assess accuracy, it appeared that the wearable camera images provided better information for differentiating background screens from screens being actively engaged with, as well as providing a closer and more detailed image that could enable assessment of content. In contrast, the room video images appeared to provide better information when the child was moving or prone or lying, when the interaction with technology was brief or involved hand controls, the context of use that could include course, and the use of multiple devices.

Coding issues were identified related to wearable camera and room video differences, including sampling rate, camera movement, and field of view.

#### Examples of Coding Issues Related to Differing Sampling Rates

**Figure 2.**
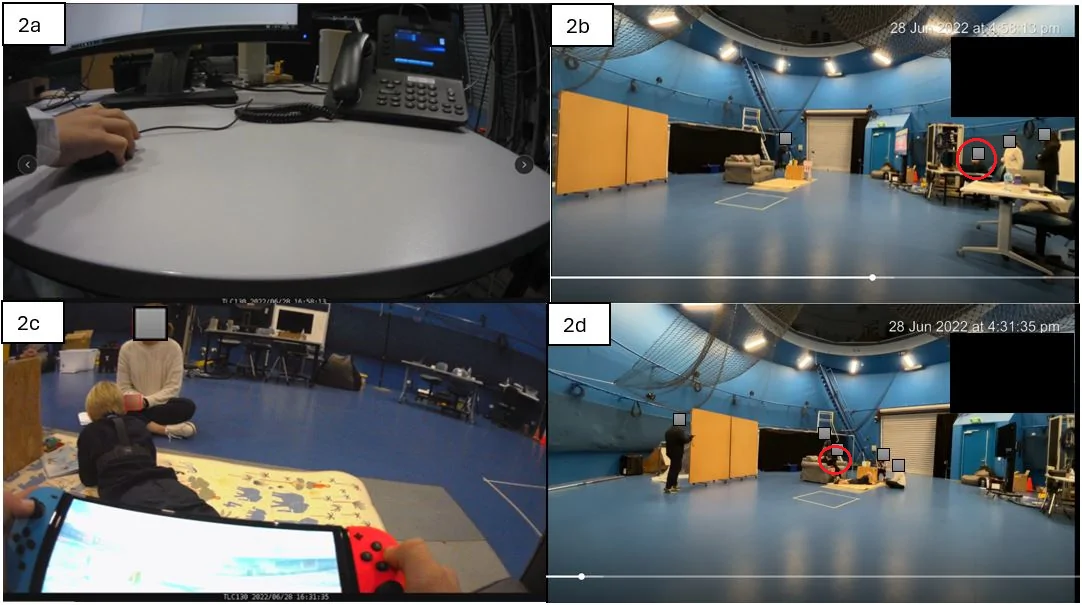
Impact of sampling rate on coding of longer duration activities. (A) Wearable camera coded desktop, (B) room video coded desktop, (c) wearable camera coded handheld gaming, and (D) room video coded handheld gaming. The red circles indicate the child with the wearable camera.

The highest agreement when considering whether a technology was coded was found for more stationary technology use or technology held closer to the child for a longer duration of time; for example, “any desktop*”* (8784/12,628, 69.6%) had fair agreement (PABAK: 0.391), and “any handheld gaming console” (6314/11,705, 53.9%) had slight agreement (PABAK: 0.079). Figure 2 shows instances where a desktop and a handheld gaming console were coded in agreement based on both the wearable camera image and room video frame.

**Figure 3.**
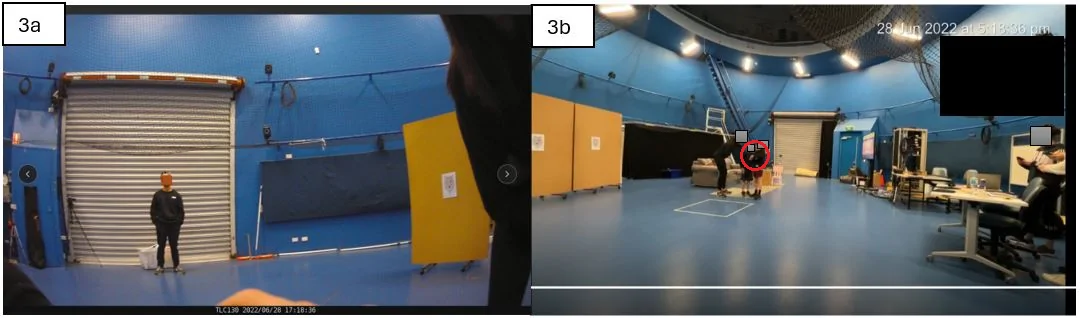
Impact of sampling rate on coding of brief activity. (A) Wearable camera coded no digital technology. (B) Room video coded smartwatch. Red circle indicates the child with the wearable camera.

All other technologies were rated as having below-chance agreement, with the lowest agreement for coding of “any smartwatch” (86/2821, 3%, PABAK: −0.939), which was typically a very brief activity. Smartwatch was coded over an order of magnitude more frequently based on the room video (2661/182,451, 1.5%) compared to the wearable camera image (246/182,451, 0.1%; Table 4). Figure 3 shows an instance when “smartwatch” was coded based on the room video, but “no digital technology” was coded based on the wearable camera.

#### Examples of Coding Issues Related to Camera Movement

**Figure 4.**
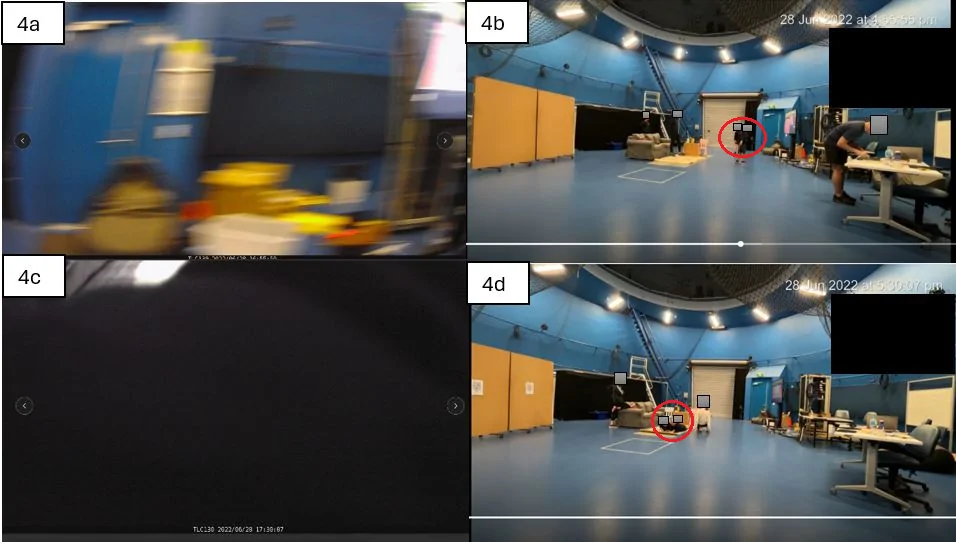
Effect of camera movement or position. (A) Wearable camera uncodable: blurry, (B) room video coded single controller and television, (C) wearable camera uncodable: obscured image, and (D) room video coded smartphone. Red circles indicate the child with the wearable camera.

*“*Uncodable” was coded more frequently based on wearable camera images (17,802/182,451, 9.8%) than on room video frames (n=3, 0%). Likewise, more wearable camera images (n=234, 0.1%) than room video frames (n=39, 0.02%) were coded as “unsure.” The reason for wearable camera images to be coded as “uncodable” was likely often due to camera movement or a slower shutter speed causing images to be too blurry to code or due to the camera being obscured (eg, child lying on their stomach). Figure 4 shows instances when the wearable camera image was coded as “uncodable” due to being blurry or obscured, but technology was coded based on the room video frame.

#### Examples of Coding Issues Related to Differing Fields of View

**Figure 5.**
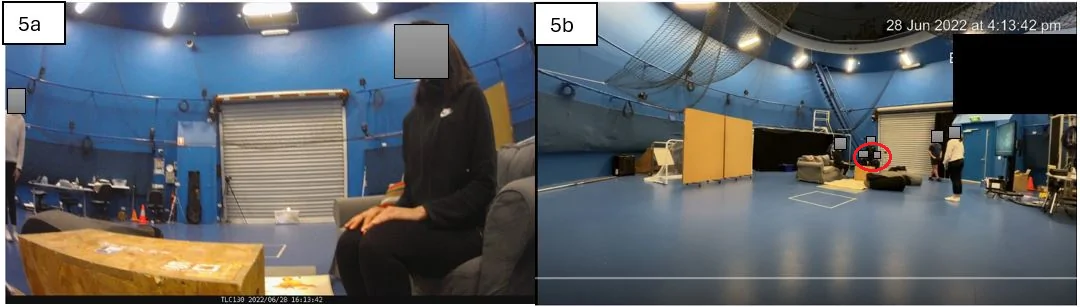
Impact of differing fields of view. (A) Wearable camera coded no digital technology and (B) room video coded television. Red circles indicate the child with the wearable camera.

Overall, a higher number of frames were coded as “no digital technology” based on the wearable camera images (84,178/182,451, 46.1%) compared to room video frames (52,504/182,451, 28.8%). Accordingly, percent agreement between wearable camera images and room video frames coded as “no digital technology” was only 48.9% (n=41,113). If a wearable camera image was coded as “no digital technology,” it was frequently coded as containing a technology based on the room video frame, particularly “television” (8783/84,178, 10.4%), “laptop” (7866/84,178, 9.3%), or “tablet” (6387/84,178, 7.6%). However, if a room video image was coded as “no digital technology,” it was only infrequently coded as a technology based on wearable camera images. Figure 5 shows instances when “no digital technology” was coded based on the wearable camera but “television” was coded based on the room video.

#### Examples of Coding Issues Related to Technology Device Differences

To identify patterns of differences in classifications between wearable camera image and room video frame coding and to determine if a technology (ie, television) was coded as different between the wearable camera and room video or in combination with other technology types, the 53-category confusion matrix can be considered. Multimedia Appendix 3 contains a link to an interactive version of the 53-category confusion matrix, along with an explanation of how to use the interactive figure and clarification of where the link will take the reader.

**Figure 6.**
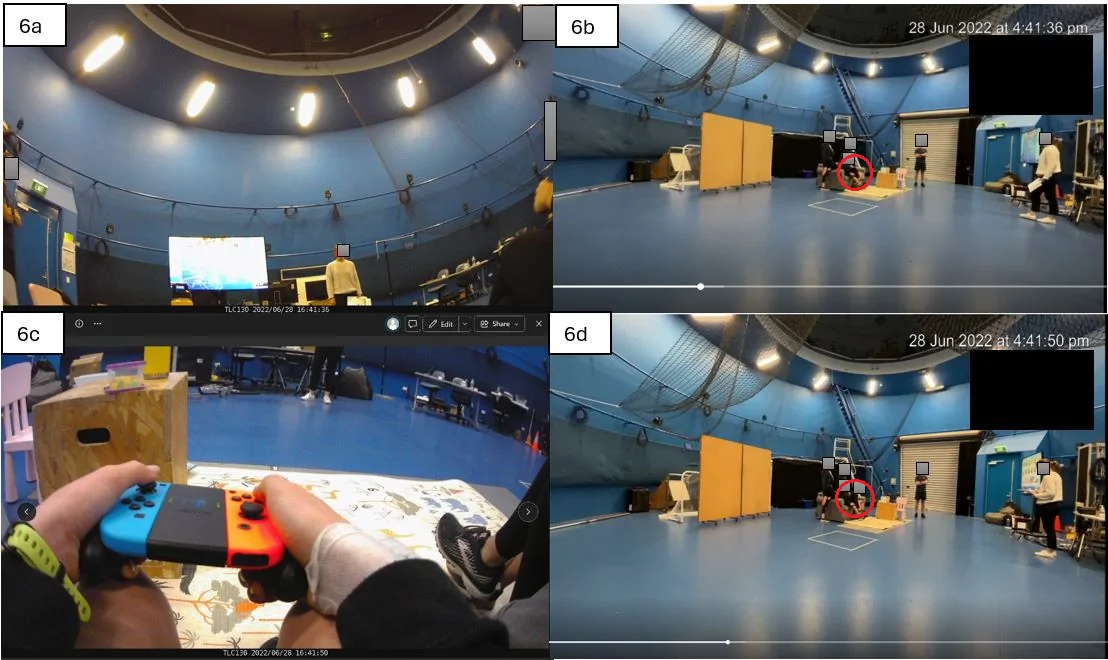
Impact of field of view and thus ability to code from gaming involving hand controllers and television screen. (A) Wearable camera coded television, (B) room video coded combined controller and television, (C) wearable camera coded combined controller, and (D) room video coded combined controller and television. Red circles indicate the child with the wearable camera.

On the basis of the 53-category confusion matrix, 3825 image and frame were coded as “television” by both the wearable camera and room video (percent agreement: 17.5%). When considering images coded as “television” based on the wearable camera (21,900/182,451, 12%), 8161 (37%) frames were coded as “television & single gaming controller” based on the room video, and 3579 (16%) frames were coded as “television & combined gaming controller*”* based on the room video. When considering frames coded as “television” based on the room video recording (18,266/182,451, 10%), there were low numbers of images coded as “television & single gaming controller” (219/18,266, 1.2%) or “television & combined gaming controller*”* (206/18,266, 1.1%) based on the wearable camera. A total of 3028 images per frame were coded based on both the wearable camera and room video as “television & combined gaming controller” (percent agreement: 32.4%), and a total of 1559 images per frame were coded based on both the wearable camera and room video as “television & single gaming controller” (percent agreement: 9.7%). Figure 6 shows instances when only “television” or “combined gaming controller*”* were coded based on the wearable camera image, but “television & combined gaming controller” was coded based on the room video frame.

**Figure 7.**
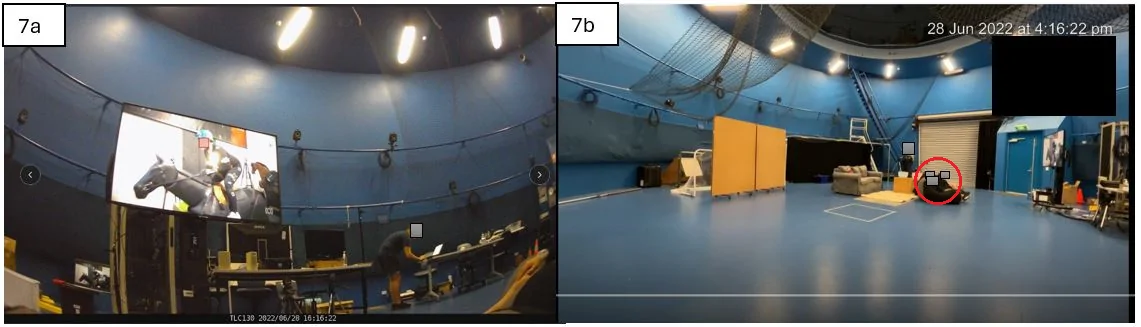
Impact of field of view on capturing television and laptop technology. (A) Wearable camera coded television and laptop and (B) room video coded television and laptop. Red circle indicates the child with the wearable camera.

“Television” as coded based on the room video (18,266/182,451, 10%) was often coded as “television & laptop” (1955/18,266, 10.7%) based on wearable camera. Similarly, “television” as coded based on the wearable camera (21,900/182,451, 12.0%) was also often coded “television & laptop” (1064/21,900, 4.9%) based on the room video. A total of 4068 images per frame were coded based on both the wearable camera and room video as “television & laptop,” with a percent agreement of 40.8%. Figure 7 shows an instance when both “television & laptop” were coded based on both the wearable camera image and the room video frames, highlighting the level of detail visible.

### Gender and Age Group Stratification

There were similar frequencies of codes between genders and age groups for the 9 “any technology combination” coded based on the wearable camera and room video (Table S3 in Multimedia Appendix 1). Overall, there were no significant differences in the PABAK results between either gender or age groups (Table S3 in Multimedia Appendix 1).

## Discussion

### Summary of Results

Overall, there were generally very low levels of agreement between the codes based on wearable camera images and room video frames for all technology types, as well as for coding whether or not technology was being used. Across all the categories, the highest agreement was found for “desktop” and “handheld gaming console” codes, and the lowest agreement was found for “smartwatch.” Differences mostly occurred when multiple technologies were being used or coded or not, as combinations of technology were often captured with the room video but not necessarily with the wearable camera. Similar patterns to those observed in the whole sample were seen when comparing genders and age groups.

Previous literature has compared self-reported and objective measurements of technology use [35,36], with a previous systematic review finding discrepancies between device-logged and self-reported digital media use and reporting that self-reported measures were rarely accurate reflections of device-logged media use [37]. While there are now many studies that have used visual images to measure human behavior, no previous studies that have directly compared wearable camera images and room video frames to understand what can be captured from each of these data sources were identified. The wearable camera and room video varied in aspects such as sampling rate, the ability of the camera to move, and field of view, leading to differences in what was captured by each source. Prior studies have compared different types of cameras and camera views for other outcomes, for example, comparing a head-worn camera with a laptop camera to assess eye tracking [38] and a head-worn camera with an overhead drone camera to assess park quality [39]. The closest identified comparison to this study compared measures of parent-infant behavior using the same head-worn video cameras on the infant, parent, and observing researcher [40]. This study found that both the direction of the field of view (eg, infant’s perspective and mother’s perspective) and the viewing distance (ie, researcher perspective vs infant and mother) influenced what could be measured. Their findings thus concur with this study that while there was concordance on some outcomes, different camera views provided better options for different outcomes. For example, infant facial expressions were best measured from the mother-worn camera; however, due to the differing visual field, whole-body movements were often missed by the first-person cameras (mother- and infant-worn camera) compared to the third-person cameras (researcher perspective) [40].

The highest agreement for technology types was observed for technology devices that were used when the child was more stationary or when the device was closer to the child (ie, desktop and handheld gaming consoles; Figure 2). Brief technology activities (eg, smartwatch use) were captured better with the room video due to higher sampling rate, resulting in the lowest agreement (Figure 3). Child engagement with a smartwatch tended to occur quickly and was therefore easily missed with the 1-second sampling frequency of camera images and may also have been missed in camera images because of blurring due to the quick movement. Smartwatches are becoming increasingly available to children; therefore, it would be important to capture use effectively [41]. In addition, when a child looked at their wrist, it may not be in view of the wearable camera (eg, the child’s arm blocking the camera). Due to these multiple issues, the use of the smartwatch may not have been captured in the wearable camera, which could perhaps be better captured in a room video recording at 30 frames per second.

Activities involving child movement (eg, jumping, running, skipping, and cartwheels) were not captured as well by the wearable camera compared to the room video. A higher percentage of images were coded as *“uncodable”* for the wearable camera images compared to the room video frames. This was often due to movement of the child or camera and a slow shutter speed, leading to blurred images. Furthermore, activities involving prone postures were not captured well by the wearable camera, as the child blocked the wearable camera view (ie, child lying on their stomach; Figure 4).

Owing to the differing fields of view, the wearable camera could miss technology but was more likely to detect active use of technology. It is likely that background use of certain technologies (ie, television and laptop) was coded based on the room video recording but not by the wearable camera images. More frames were coded as “no digital technology” based on the wearable camera images compared to the room video recording. Either the wearable camera missed capturing the technology or the room video “overcaptured” technology use. As captured by the room video, technology devices could still have been used, but due to the field of view of the wearable camera, the technology was missed. However, when determining active technology use based on the room video recording, it was sometimes hard to determine if the child was actively using or engaged with the technology or not, for example, if a technology was in the peripheral vision of the child (Figure 5). Wearable cameras primarily capture first-person, action-proximal engagement, whereas room video captures environmental availability and background exposure.

Technology use involving simultaneous use of hand gaming controllers and a television screen was not captured as well by the wearable cameras due to the field of view missing either the controller or television screen. Single and combined gaming controllers were often coded simultaneously with television for the room video frames but often only coded as “television” based on the wearable camera images (Figure 6). If single and combined gaming controllers were simultaneously coded with television in the wearable camera, then they were likely also simultaneously coded in the room video. The single and combined gaming controllers were often held outside the field of view of the wearable camera and, when within the field of view, could appear blurry in the wearable camera images due to movement of the child and controllers and a slow shutter speed. On the basis of the wearable camera, it was sometimes difficult to determine whether the child was simply watching television or gaming using the television, whereas this could be captured within the room video given the different point of view.

### Pros and Cons of Each Data Source

Room video data had a wider field of view; therefore, it was able to better capture technology that was in the room and had the ability to capture the context of the technology use. With the ability to capture the whole room, it was clear when the child was passively watching a program on the television compared to actively playing a video game. Additionally, although not an aim of this study, room video would have the ability to capture coviewing and co-use of technology (as seen in Figures 2–7). Borzekowski and Robinson [16] previously used a fixed room camera to capture coviewing of television and behaviors while watching television, such as eating, reading, or doing homework, and playing. On the other hand, the wider field of view of the video recording sometimes limited the quality of the video frames, and therefore, on occasion, it was hard to determine if there was technology present (eg, a laptop in the background). In addition, although not an aim of this study, it would be difficult to determine the content of the technology. For example, on occasion, it was difficult to determine if the television was actively on with a program or just a standby screen; therefore, it would be difficult to determine if the content was a violent movie compared to educational children’s content. Furthermore, when determining active technology use based on the room video, it was sometimes difficult to determine if the child was actively using or engaged with the technology or not, for example, capturing technology in the peripheral vision of the child (as seen in Figure 5). Vadathya et al [21] previously attempted to combat this issue by developing a method to determine whether the target child was looking at the television using a home-based fixed room camera. There are also privacy concerns with recording room images.

The wearable camera had a smaller field of view but was closer in proximity and more focused (as seen in Figures 2 and 7). The wearable camera was able to capture active engagement with the technology and, due to the proximity to the technology, would have a greater ability to capture the content on the screen (as seen in Figure 7). Previous studies using wearable cameras to measure device use by adolescents have shown that it is feasible to code multiple screens with details of the screen content (eg, gaming, television viewing, social media, communication, creative content, and internet) as well as the location (eg, bedroom and living room) [18,19]. However, a wearable camera also has limitations. The children were often moving, which led to blurry images (as seen in Figure 4). With the camera mounted on the chest, sometimes some technologies were not visible either due to the position of the child or the placement of the technology (as seen in Figure 6). Particularly for gaming, the controller was frequently not visible in the wearable camera frame due to the child holding the controller out of view or due to movement of the child (such as using the single controller during active play). Therefore, the task may have been missed, with both gaming and television watching activities being coded as “television.” Sometimes only a very small part of the screen was visible (eg, the corner of a tablet), therefore potentially missing technology that was out of frame. Moreover, if the engagement with the technology occurred quickly (eg, using a smartwatch), it was easily missed with images only captured every second (as seen in Figure 3). Furthermore, if the child was in certain positions (eg, lying on their stomach), the camera could be blocked, leading to “uncodable” images (as seen in Figure 4). Previous studies have also found that wearable cameras have the ability to capture screen use when the participant is upright or partially upright but capture a high percentage of images of the ceiling or obscure images due to the participants being positioned on their backs or in other positions [18,42,43]. Despite an ethical framework being established for privacy concerns with wearable cameras [28], parents remained concerned, especially with the varied locations of use creating greater potential for capture of nonconsenting third parties. The advantages and challenges of each data source are summarized in Table 5.

**Table 5.** Advantages and challenges of fixed room video and wearable cameras.

Method	Advantages	Challenges
Fixed room video	Can capture technology within the whole space Can capture the context of the technology, such as passively watching television or actively playing a video game Can capture coviewing and co-use of technology	Difficult to determine content on technology device Difficult to determine active engagement with technology compared to background use Privacy concerns with recording room images
Wearable camera	Can capture active engagement with the technology Has the ability to capture the content on the screen Has the ability to capture technology use in various locations	Movement can result in blurry images Technology may not be visible within field of view Privacy concerns with the ability to capture content in varied locations

### Strengths and Limitations

A strength of this study was the simultaneous use of both a wearable camera and 2 fixed room video cameras to capture technology use. Another strength was that there was the broad age range from 3 to 14 years and the wide variety of technology devices included in the study. However, there were several limitations. First, the video camera placement at approximately 6 m from the center of the laboratory led to reduced video frame quality. There was also a potential that the time stamp synchronization of the wearable camera images and room video frames was not perfect and that small discrepancies in the time stamp synchronization could result in mismatches, which would affect the agreement of brief activities, such as checking the smartwatch. Finally, as the data were collected in a laboratory setting, the results should be generalized with caution to free-living conditions. We encourage further research in free-living conditions, despite the challenges of such studies.

### Implications for Further Research

If future research is continued in free-living conditions, a few considerations should be made. Similar to previous literature [28], caregivers expressed concerns about privacy if the devices were used in a home setting and were worried about other children being captured if the devices were used in a school setting. Caregivers were more comfortable if the recording devices could be turned on and off and if the devices could be smaller. There were also concerns from caregivers about wearing the camera during certain activities, such as contact sports and swimming.

The coding of the wearable camera and fixed video frames was based on decisions made by the coders. In practice, researchers are likely to be able to interpolate coding for some blurred or obscured images, for example, when a screen temporarily moves out of view. This approach was not used in this study to enable clear comparisons of issues, such as movement blurring. Coding of both sources had the potential for human error and was extremely time-consuming (taking ~6 h per hour of recorded data), which suggests that it may be unsuitable for large-scale studies. Machine learning software could potentially be used to reduce the high researcher burden for the analysis of technology use via camera images or video frames [7], and advances in AI should be examined for accuracy in coding child behaviors from images [44].

Given the complexity of modern technology use, future research could usefully evaluate the simultaneous use of several data collection methods to use the relative advantages of different methods [7]. This could include questionnaires to provide some complementary information for image data, such as context or purpose. Images from both the wearable camera and the stationary video camera can capture useful and complementary information and thus could be used together. Similarly, multiple objective measures could be used simultaneously, for example, combining on-device recording of apps used or websites visited to triangulate the classification of content coded from image data.

### Conclusions

Overall, there was poor agreement for children’s technology use between the wearable camera images and room video frames. The results did not differ based on gender or age of the participants. The wearable camera and room video varied in sampling rate, the ability to move, and perspective, leading to differences in what was captured by each method. Both the room video and wearable camera methods have strengths and limitations due to these differences. Overall, fixed room video might better capture that technology is within a single set space and the context of the technology use, and the view and higher sampling rate of video may be better when child movement or prone lying is involved, handheld devices are used, interactions with technology are brief, or multiple devices are used within a set location. Wearable cameras could better differentiate the active use of technology versus background television, the content of the technology (due to the closer view of screen detail), and different locations of use. Future use of wearable cameras and room video to capture digital technology use should carefully consider the purpose of the study along with the potential limitations of each data source. Capturing digital technology use by children is difficult, with no gold standard and no one ideal method.

## Supplementary material

10.2196/91113 Multimedia Appendix 1 Supplementary tables including technology coding categories, frequency and agreement of technology coding, and gender and age-group stratification.

10.2196/91113 Multimedia Appendix 2 Consort style figure of flow of participants and data.

10.2196/91113 Multimedia Appendix 3 Interactive confusion matrix: instructions and interpretation.

## Abbreviations

PABAK: prevalence-adjusted bias-adjusted κ
