# Supplementary material for: Comparison of Capturing Children’s Technology Use Using Wearable Camera and Fixed Room Video: Experimental Laboratory Study
Source: JMIR Pediatr Parent. 2026 Sep 1;9:e91113. doi: 10.2196/91113 (PMC13532691; doi:10.2196/91113)
Supplement: Supplementary file 1 [file pediatrics-v9-e91113-s001.docx]

**Table S1: Technology coding categories**

|  | **Technology coding categories (N=53)** |
| --- | --- |
| Uncodable or unsure (n=2) | Uncodable (Blurry, obscured or poor lighting) |
|  | Unsure |
| No digital technology (n=1) | No digital technology |
| One technology (n=9) | Television |
|  | Desktop |
|  | Laptop |
|  | Tablet |
|  | Smartphone |
|  | Smartwatch |
|  | Handheld gaming |
|  | Combined controller |
|  | Single controller |
| Two technologies (n=26) | Combined controller & combined controller |
|  | Combined controller & handheld gaming |
|  | Desktop & single controller |
|  | Desktop & smartwatch |
|  | Laptop & desktop |
|  | Laptop & combined controller |
|  | Laptop & handheld gaming |
|  | Laptop & laptop |
|  | Laptop & single controller |
|  | Laptop & smartwatch |
|  | Single controller & single controller |
|  | Smartphone & smartphone |
|  | Smartwatch & smartphone |
|  | Tablet & desktop |
|  | Tablet & single controller |
|  | Tablet & smartphone |
|  | Tablet & smartwatch |
|  | Tablet & tablet |
|  | Television & combined controller |
|  | Television & desktop |
|  | Television & handheld gaming |
|  | Television & laptop |
|  | Television & single controller |
|  | Television & smartphone |
|  | Television & smartwatch |
|  | Television & tablet |
| Three technologies (n=15) | Tablet & single controller & single controller |
|  | Tablet & tablet & tablet |
|  | Television & combined controlled & combined controller |
|  | Television & handheld gaming & combined controller |
|  | Television & laptop & combined controller |
|  | Television & laptop & single controller |
|  | Television & laptop & smartphone |
|  | Television & laptop & smartwatch |
|  | Television & single controller & single controller |
|  | Television & single controller & smartphone |
|  | Television & single controller & tablet |
|  | Television & smartphone & smartphone |
|  | Television & tablet & smartphone |
|  | Television & tablet & smartwatch |
|  | Television & tablet & tablet |

**Table S2: Frequency and agreement of technology coding based on wearable camera and room video recordings.**

| **Technology coded n (%)** | **Wearable camera images  (n (%))** | **Room video frames**  **(n (%))** | **Agreement**  **(n (%))** |
| --- | --- | --- | --- |
| Uncodable | 17802 (9.8%) | 3 (0%) | 0 (0%) |
| Unsure | 234 (0.1%) | 39 (0.02%) | 0 (0%) |
| No digital technology | 84178 (46.1%) | 52504 (28.8%) | 41113 (48.9%) |
| Television | 21900 (12.0%) | 18266 (10.0%) | 3825 (17.5%) |
| Desktop | 9426 (5.2%) | 11893 (6.5%) | 8759 (73.6%) |
| Laptop | 11596 (6.4%) | 19150 (10.5%) | 10510 (54.9%) |
| Tablet | 7327 (4.0%) | 14565 (8.0%) | 5299 (36.4%) |
| Smartphone | 4866 (2.7%) | 9120 (5%) | 3063 (33.5%) |
| Smartwatch | 182 (0.1%) | 1822 (1%) | 57 (3.1%) |
| Handheld gaming | 6812 (3.7%) | 10758 (5.9%) | 5988 (55.7%) |
| Combined controller | 1922 (1.1%) | 486 (0.3%) | 182 (9.4%) |
| Single controller | 176 (0.1%) | 30 (0.02%) | 0 (0%) |
| Combined controller & combined controller | 12 (0.01%) | 0 (0%) | 0 (0%) |
| Combined controller & handheld gaming | 21 (0.01%) | 0 (0%) | 0 (0%) |
| Desktop & single controller | 1 (0%) | 0 (0%) | 0 (0%) |
| Desktop & smartwatch | 0 (0%) | 34 (0.02%) | 0 (0%) |
| Laptop & desktop | 4 (0%) | 0 (0%) | 0 (0%) |
| Laptop & combined controller | 76 (0.04%) | 0 (0%) | 0 (0%) |
| Laptop & handheld gaming | 310 (0.2%) | 113 (0.1%) | 75 (24.2%) |
| Laptop & laptop | 1 (0%) | 0 (0%) | 0 (0%) |
| Laptop & single controller | 8 (0%) | 0 (0%) | 0 (0%) |
| Laptop & smartwatch | 0 (0%) | 59 (0.03%) | 0 (0%) |
| Single controller & single controller | 42 (0.02%) | 0 (0%) | 0 (0%) |
| Smartphone & smartphone | 84 (0.05%) | 0 (0%) | 0 (0%) |
| Smartwatch & smartphone | 0 (0%) | 50 (0.03%) | 0 (0%) |
| Tablet & desktop | 49 (0.03%) | 0 (0%) | 0 (0%) |
| Tablet & single controller | 1 (0%) | 0 (0%) | 0 (0%) |
| Tablet & smartphone | 6 (0%) | 262 (0.1%) | 1 (0.4%) |
| Tablet & smartwatch | 0 (0%) | 76 (0.04%) | 0 (0%) |
| Tablet & tablet | 1111 (0.6%) | 0 (0%) | 0 (0%) |
| Television & combined controller | 4142 (2.3%) | 9346 (5.1%) | 3028 (32.4%) |
| Television & desktop | 0 (0%) | 9 (0%) | 0 (0%) |
| Television & handheld gaming | 10 (0.01%) | 0 (0%) | 0 (0%) |
| Television & laptop | 6608 (3.6%) | 9970 (5.5%) | 4068 (40.8%) |
| Television & single controller | 2034 (1.1%) | 16005 (8.8%) | 1559 (9.7%) |
| Television & smartphone | 460 (0.3%) | 4117 (2.3%) | 150 (3.6%) |
| Television & smartwatch | 64 (0.04%) | 432 (0.2%) | 8 (1.8%) |
| Television & tablet | 389 (0.2%) | 3147 (1.7%) | 155 (5.0%) |
| Tablet & single controller & single controller | 1 (0%) | 0 (0%) | 0 (0%) |
| Tablet & tablet & tablet | 6 (0%) | 0 (0%) | 0 (0%) |
| Television & combined controlled & combined controller | 45 (0.02%) | 0 (0%) | 0 (0%) |
| Television & handheld gaming & combined controller | 1 (0%) | 0 (0%) | 0 (0%) |
| Television & laptop & combined controller | 95 (0.05%) | 0 (0%) | 0 (0%) |
| Television & laptop & single controller | 88 (0.05%) | 0 (0%) | 0 (0%) |
| Television & laptop & smartphone | 30 (0.02%) | 0 (0%) | 0 (0%) |
| Television & laptop & smartwatch | 0 (0%) | 171 (0.09%) | 0 (0%) |
| Television & single controller & single controller | 286 (0.2%) | 0 (0%) | 0 (0%) |
| Television & single controller & smartphone | 1 (0%) | 0 (0%) | 0 (0%) |
| Television & single controller & tablet | 3 (0%) | 0 (0%) | 0 (0%) |
| Television & smartphone & smartphone | 32 (0.02%) | 0 (0%) | 0 (0%) |
| Television & tablet & smartphone | 0 (0%) | 13 (0.01%) | 0 (0%) |
| Television & tablet & smartwatch | 0 (0%) | 11 (0.01%) | 0 (0%) |
| Television & tablet & tablet | 9 (0%) | 0 (0%) | 0 (0%) |

**Tables 3: Gender and age-group stratification**

**Boys (N=19)**

| **Technology** | **Camera images (n)** | **Video  (n)** | **Total N*** | **Agreement  (n (%))** | **PABAK** |
| --- | --- | --- | --- | --- | --- |
| Any television | 14628 | 27644 | 29797 | 12475 (41.9%) | -0.163 |
| Any desktop | 4091 | 5191 | 5574 | 3708 (66.5%) | 0.330 |
| Any laptop | 7252 | 11831 | 13184 | 5899 (44.7%) | -0.105 |
| Any tablet | 3493 | 7775 | 8139 | 3129 (38.4%) | -0.231 |
| Any smartphone | 2648 | 6571 | 6952 | 2267 (32.6%) | -0.348 |
| Any smartwatch | 129 | 1316 | 1388 | 57 (4.1%) | -0.918 |
| Any handheld gaming | 2686 | 4755 | 4947 | 2494 (50.4%) | 0.008 |
| Any combined controller | 2576 | 4371 | 4823 | 2124 (44.0%) | -0.119 |
| Any single controller | 1202 | 7771 | 8033 | 940 (11.7%) | -0.766 |

**Girls (N=25)**

| **Technology** | **Camera images (n)** | **Video  (n)** | **Total N*** | **Agreement  (n (%))** | **PABAK** |
| --- | --- | --- | --- | --- | --- |
| Any television | 21570 | 33833 | 37820 | 17583 (46.5%) | -0.070 |
| Any desktop | 5388 | 6742 | 7054 | 5076 (72.0%) | 0.439 |
| Any laptop | 11564 | 17630 | 19893 | 9301 (46.8%) | -0.065 |
| Any tablet | 5411 | 10300 | 11011 | 4700 (42.7%) | -0.146 |
| Any smartphone | 2832 | 6993 | 7336 | 2489 (33.9%) | -0.321 |
| Any smartwatch | 117 | 1345 | 1433 | 29 (2.0%) | -0.960 |
| Any handheld gaming | 4468 | 6110 | 6758 | 3820 (56.5%) | 0.131 |
| Any combined controller | 3738 | 5458 | 6332 | 2864 (45.2%) | -0.095 |
| Any single controller | 1440 | 8262 | 8759 | 943 (10.8%) | -0.785 |

**Test (either t-test or Wilcoxon depending on distribution of data) comparing PABAK between genders:**

| **Technology** | **p-value** | **Method** |
| --- | --- | --- |
| Any television | 0.38 | Two Sample t-test |
| Any desktop | 0.22 | Wilcoxon |
| Any laptop | 0.31 | Two Sample t-test |
| Any tablet | 0.45 | Two Sample t-test |
| Any smartphone | 0.09 | Two Sample t-test |
| Any smartwatch | 0.72 | Wilcoxon |
| Any handheld gaming | 0.78 | Wilcoxon |
| Any combined controller | 0.86 | Two Sample t-test |
| Any single controller | 0.18 | Two Sample t-test |

**Age 3-5 (n=11)**

| **Technology** | **Camera images (n)** | **Video  (n)** | **Total N*** | **Agreement  (n (%))** | **PABAK** |
| --- | --- | --- | --- | --- | --- |
| Any television | 7974 | 13034 | 14598 | 6410 (43.9%) | -0.122 |
| Any desktop | 1986 | 2403 | 2578 | 1811 (70.2%) | 0.405 |
| Any laptop | 4840 | 7558 | 8287 | 4111 (49.6%) | -0.008 |
| Any tablet | 2234 | 4319 | 4655 | 1898 (40.8%) | -0.185 |
| Any smartphone | 878 | 2080 | 2285 | 673 (29.5%) | -0.411 |
| Any smartwatch | 8 | 584 | 585 | 7 (1.2%) | -0.976 |
| Any handheld gaming | 1491 | 2656 | 2858 | 1289 (45.1%) | -0.098 |
| Any combined controller | 1172 | 2025 | 2257 | 94 (41.6%) | -0.167 |
| Any single controller | 414 | 2709 | 2851 | 272 (9.5%) | -0.809 |

**Age 5-10 (n=22)**

| **Technology** | **Camera images (n)** | **Video  (n)** | **Total N*** | **Agreement  (n (%))** | **PABAK** |
| --- | --- | --- | --- | --- | --- |
| Any television | 19054 | 34483 | 37752 | 15785 (41.8%) | -0.164 |
| Any desktop | 5169 | 6383 | 6742 | 4810 (71.3%) | 0.427 |
| Any laptop | 9329 | 14873 | 16721 | 7481 (44.7%) | -0.105 |
| Any tablet | 4286 | 8803 | 9221 | 3868 (41.9%) | -0.161 |
| Any smartphone | 3193 | 8217 | 8618 | 2792 (32.4%) | -0.352 |
| Any smartwatch | 177 | 1660 | 1775 | 62 (3.5%) | -0.93 |
| Any handheld gaming | 3750 | 5470 | 5737 | 3483 (60.7%) | 0.214 |
| Any combined controller | 4211 | 5211 | 6164 | 3258 (52.9%) | 0.057 |
| Any single controller | 1534 | 9639 | 10037 | 1136 (11.3%) | -0.774 |

**Age 11-14 (n=11)**

| **Technology** | **Camera images (n)** | **Video  (n)** | **Total N*** | **Agreement  (n (%))** | **PABAK** |
| --- | --- | --- | --- | --- | --- |
| Any television | 9170 | 13960 | 15267 | 7863 (51.5%) | 0.03 |
| Any desktop | 2324 | 3147 | 3308 | 2163 (65.4%) | 0.308 |
| Any laptop | 4647 | 7030 | 8069 | 3608 (44.7%) | -0.106 |
| Any tablet | 2384 | 4953 | 5274 | 2063 (39.1%) | -0.218 |
| Any smartphone | 1409 | 3267 | 3385 | 1291 (38.1%) | -0.237 |
| Any smartwatch | 61 | 417 | 461 | 17 (3.7%) | -0.926 |
| Any handheld gaming | 1913 | 2739 | 3110 | 1542 (49.6%) | -0.008 |
| Any combined controller | 931 | 2593 | 2734 | 790 (28.9%) | -0.422 |
| Any single controller | 694 | 3685 | 3904 | 475 (12.2%) | -0.757 |

**Test (either ANOVA or Kruskal-Wallis depending on distribution of data) comparing PABAK between age-groups:**

| **Technology** | **p-value** | **Method** |
| --- | --- | --- |
| Any television | 0.14 | ANOVA |
| Any desktop | 0.37 | Kruskal-Wallis |
| Any laptop | 1.00 | ANOVA |
| Any tablet | 0.15 | ANOVA |
| Any smartphone | 0.06 | ANOVA |
| Any smartwatch | 0.67 | Kruskal-Wallis |
| Any handheld gaming | 0.31 | Kruskal-Wallis |
| Any combined controller | 0.25 | ANOVA |
| Any single controller | 0.09 | Kruskal-Wallis |
