# Supplementary material for: Comparison of Capturing Children’s Technology Use Using Wearable Camera and Fixed Room Video: Experimental Laboratory Study
Source: JMIR Pediatr Parent. 2026 Sep 1;9:e91113. doi: 10.2196/91113 (PMC13532691; doi:10.2196/91113)
Supplement: Supplementary file 2 [file pediatrics-v9-e91113-s002.docx]

**Supplementary File S1.**

*Consort style figure of flow of participants and data*

Children completing activities (n=48)

Children missing usable wearable camera images (n=4)

**Collected**

Children with both wearable camera and room video data

(n = 44)

**Included**

Data included in study

Wearable camera images (n=182,451; 50.7hrs)

Room video images

(n=182,451; 50.7hrs)
