# Supplementary material for: Comparison of Capturing Children’s Technology Use Using Wearable Camera and Fixed Room Video: Experimental Laboratory Study
Source: JMIR Pediatr Parent. 2026 Sep 1;9:e91113. doi: 10.2196/91113 (PMC13532691; doi:10.2196/91113)
Supplement: Supplementary file 3 [file pediatrics-v9-e91113-s003.docx]

*Interactive confusion matrix: instructions and interpretation*

The interactive version of the confusion matrix comparing wearable camera and room video classifications across 53 technology categories is available at: <https://rpubs.com/DrCLR/cm_53_categories_camera_video>

The link directs to a hosted visualization (RPubs), a publicly accessible platform developed by Posit (a Public-Benefit Corporation founded by a successful USA programmer) for sharing reproducible analysis and data visualisations ([https://rpubs.com](https://rpubs.com/)). The content published on Rpubs is accessible via a standard web browser and does not require user authentication or software installation.

*How to interpret the figure*

- Columns represent room video classifications.
- Rows represent wearable camera classifications.
- Each cell displays the number of observations (N).
- Colour intensity reflects the proportion of observations within each room video category classified into each wearable camera category.
- Perfect agreement is represented along the diagonal of the matrix.
- Off-diagonal cells indicate disagreements.

*How to use the interactive features:*

The toolbar in the top right corner of the figure provides several tools to explore the matrix:

- Zoom (magnifying glass icon)
  Allows you to zoom into a specific region of the matrix by clicking and dragging a box over the area of interest.
- Pan (crossed arrows icon)
  Allows you to move the view around after zooming.
  *In practical terms, this means you can “drag” the figure to explore different parts without changing the zoom level.*
- Zoom in / Zoom out (+ / – icons)
  Gradually increase or decrease the level of zoom.
- Reset axes (home icon)
  Returns the figure to its original full view.
- Download as image (camera icon)
  Saves the current view of the figure as an image file.
